# Periostin Upregulates Wnt/β-Catenin Signaling to Promote the Osteogenesis of *CTLA4-*Modified Human Bone Marrow-Mesenchymal Stem Cells

**DOI:** 10.1038/srep41634

**Published:** 2017-01-27

**Authors:** Fei Zhang, Keyu Luo, Zhigang Rong, Zhengdong Wang, Fei Luo, Zehua Zhang, Dong Sun, Shiwu Dong, Jianzhong Xu, Fei Dai

**Affiliations:** 1 Department of Orthopaedics, National & Regional United Engineering Laboratory, Southwest Hospital, Third Military Medical University, Chongqing, China; 2 Department of Biomedical Materials Science, School of Biomedical Engineering, Third Military Medical University, Chongqing, China

## Abstract

The enhanced osteogenesis of mesenchymal stem cells (MSCs) modified by expression of cytotoxic T lymphocyte-associated antigen 4 (CTLA4) has been shown in previous studies, but the mechanism remains unknown. Here we found that the bone repair effect of *CTLA4*-modified MSCs in demineralized bone matrix (DBM) in a rabbit radius defect model was significantly better than that observed for unmodified MSCs in DBM or DBM alone, and the periostin (POSTN) expression in *CTLA4*-modified MSCs was significantly higher than that in unmodified MSCs both *in vivo* and *in vitro.* In addition, we also found that treatment of *CTLA4*-modified MSCs with soluble POSTN could inhibit the glycogen synthase kinase-3β activity and increase β-catenin expression through up-regulation of lipoprotein-related protein-6 phosphorylation to promote osteogenic differentiation, but blocking of integrin αvβ3, a receptor of POSTN, could suppress these effects. Our data demonstrated that POSTN expressed in response to CTLA4 can promote the osteogenesis of xenotransplanted MSCs through interaction with Wnt/β-catenin pathway.

Successful treatment of a large bone defect using a tissue engineering strategy has been achieved in clinical practice^1^,^2^. However, the limited availability of autogenic (auto-) mesenchymal stem cells (MSCs) due to individual differences in patients presents a great challenge to the further application of bone tissue engineering^3^,^4^,^5^. In addition, the immunogenicity of allogeneic (allo-) MSCs is still poorly understood, and researchers have reported that allo-MSCs can elicit an immune response in allotransplantation and cause graft failure^6^,^7^,^8^. Thus, determining how to manage the immunogenicity of donor MSCs is an imminent problem for the widespread application of bone tissue engineering strategies.

Cytotoxic T lymphocyte antigen 4 (CTLA4) has been proven to be an important co-inhibitory molecule not only in mediating T-cell anergy and apoptosis but also in increasing immune tolerance^9^. Previously, we successfully transfected MSCs with the CTLA4-Ig gene, which contains the extracellular domain of CTLA4 and the Fc segment of IgG, to construct CTLA4-expressing MSCs, and we showed that these cells possess a superior ability for osteogenic differentiation in xenotransplantation. We further demonstrated that the immune activation microenvironment reduced the expression of osteogenic markers in MSCs but not in the *CTLA4*-modified MSCs^10^, which indicated that the presence of CTLA4 maintained the osteogenic differentiation of MSCs in immune activation conditions; however, the underlying mechanisms are not clear.

In general, the successful osteogenic differentiation of MSCs is a requirement for repair of a bone defect, and the tissue microenvironment is important for the directed differentiation of MSCs. The tissue microenvironment created by osteoblasts mainly consists of extracellular matrix (ECM) and is involved in regulating cell adhesion, migration, and differentiation^11^,^12^,^13^. Recently, it has been suggested that periostin (POSTN) is not only present in the ECM but also participates in cell–ECM interactions^14^. POSTN is a 90-kDa ECM protein that was originally known as osteoblast-specific factor-2 (OSF-2). It is highly expressed in adult connective tissues such as periosteum, tendon, and periodontal ligaments^15^,^16^,^17^. Multiple variants of POSTN have been demonstrated to be expressed at high levels in the periosteum during fetal development^18^. Animal studies also showed that POSTN-deficient mice exhibit disrupted collagen fibrillogenesis in the periosteum, low bone mass, reduced cortical bone volume, and increased bone damage in response to fatigue loading injury^19^,^20^. These results suggest that POSTN is not only a structural protein of the ECM, but also plays a key role in bone formation and metabolism in morphogenesis and postnatal development.

In the present study, we hypothesized that under immune activation conditions, CTLA4 can maintain POSTN expression and promote osteogenesis of MSCs to improve the bone repair effect. To test our hypothesis, we examined the ability of *CTLA4*-modified MSCs to promote the repair of a critical-sized segmental radius defects *in vivo* through bone tissue engineering and investigated the underlying mechanism, specifically the involvement of the Wnt/β-catenin pathway. Our results may elucidate an exact mechanism for an allogenic human MSC-based bone tissue engineering strategy for the repair of large bone defects and provide a new theoretical basis for clinical treatment.

## Results

### Evaluation of repair of critical-sized segmental radius defects in rabbit model

**Figure 1:**
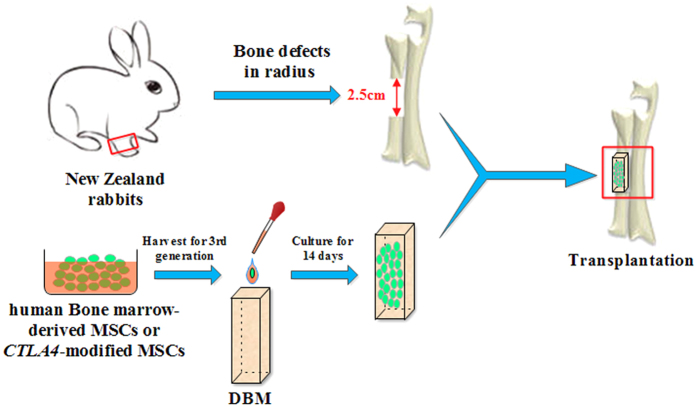
Experimental design. Critical-sized segmental radius bone defect model was created in New Zealand male rabbits (5 months old). The third passage human bone marrow-derived MSCs or *CTLA4*-modified MSCs were seeded on DBM and cultured in osteogenic medium for 14 days. Then DBM with *CTLA4*-modified MSCs, DBM withMSCs, or DBM alone was transplanted into the bone defect region (n = 6/group).

**Figure 2:**
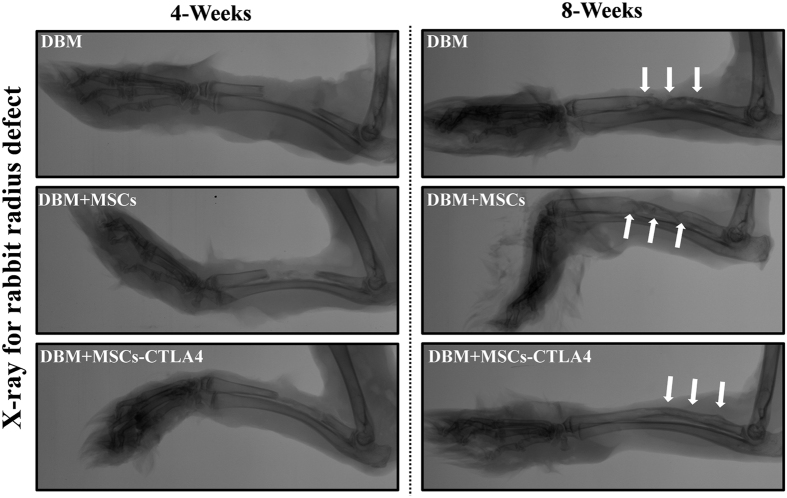
X-ray analysis of rabbit radius defect repair. X-ray images of the critical-sized segmental radius bone defects at 4 and 8 weeks after implantation. Repaired bone in the defect is indicated by arrows.

The experimental design is summarized in Fig. 1. Human bone marrow-derived MSCs and *CTLA4*-modified MSCs were prepared and then seeded onto demineralized bone matrix (DBM) to construct the tissue engineering bone by osteogenic induction for 14 days. A 2.5-cm critical-sized segmental bone defect was created in the radius of a rabbit. Then the DBM with *CTLA4*-modified MSCs, DBM with control MSCs, or DBM alone was implanted into the bone defects, and the bone repair effect was evaluated at 4 and 8 weeks postoperation. At 4 weeks, little new bone formation was observed only in the DBM with *CTLA4*-modified MSCs group. At 8 weeks, plain X-ray examination showed that osteoid tissues were presented in all implantation areas, but a better bone union was observed for the DBM with *CTLA4*-modified MSCs compared to the DBM with control MSCs or DBM alone (Fig. 2).

**Figure 3:**
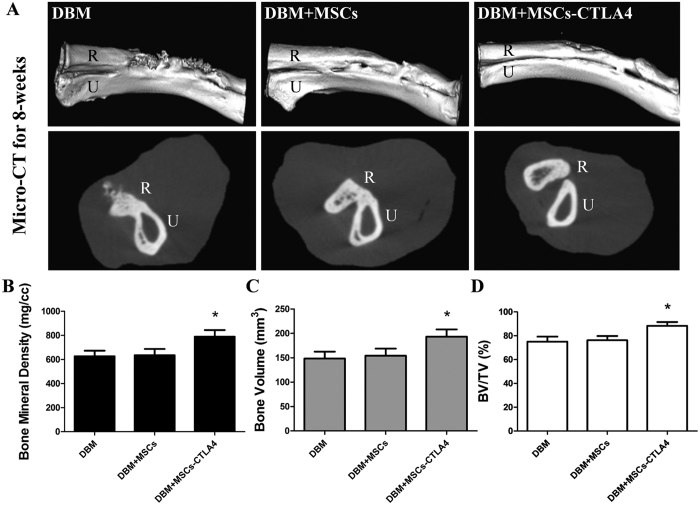
Repair of critical-sized segmental radius bone defects at 8 weeks after implantation. (**A**) Three-dimensional reconstruction of micro-CT and cross-sectional images of radius (R) and ulna (U). (**B~D**) Morphometric analyses of bone mineral density, bone volume, and bone volume fraction (BV/TV) of regenerated tissues in the defect area. *P < 0.05.

Rabbits were euthanized after 8 weeks, and the radius and ulna were analyzed by micro-computed tomography (CT) for bone formation. Our results suggested that bone defects were repaired with high-density tissue in all groups, but the degree of bone formation differed (Fig. 3A). The cross-sectional images showed that the marrow cavity of the radius was recanalized only in defects that received DBM with *CTLA4*-modified MSCs, and not in defects treated with DBM with control MSCs or in DBM alone. In addition, the bone mineral density, bone volume (BV), and bone volume fraction (BV/TV) were significantly higher for the group that received DBM with *CTLA4*-modified MSCs than in those that received DBM with control MSCs or in DBM alone (p < 0.05; Fig. 3B~D).

### Histological analysis of new bone formation and POSTN and β-catenin expression

**Figure 4:**
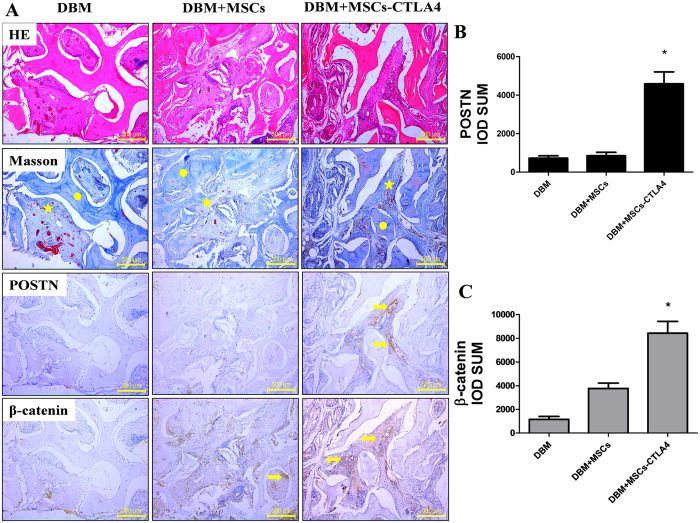
Detection of POSTN and β-catenin expression in the tissue engineering xenograft. (**A**) Histological analysis by H&E and Masson’s Trichrome staining (circle: DBM; pentagram: new bone formation area) of newly formed bone, and the expressions of POSTN and β-catenin detected by immunohistochemistry at 2 months post implantation (arrow: positive expression). Scale bars: 500 μm. (**B**,**C**) The IOD of POSTN and β-catenin was quantified by Image-Pro Plus 6.0. n = 3, *P < 0.05.

The bone tissues formed within defects at 2 months post-transplantation were examined by hematoxylin and eosin (H&E) staining and Masson Trichrome staining. Newly formed bone was observed in all groups as indicated in Fig. 4A.

Then we investigated the expression of POSTN and β-catenin through immunohistochemical staining. Our data suggest that little POSTN was expressed in tissues formed from DBM with control MSCs or DBM alone, but POSTN was highly expressed in the tissue formed by DBM with *CTLA4*-modified MSCs. Moreover, the quantitative analysis of integral optical density (IOD) showed that the POSTN expression in the area of new bone formation in the DBM with *CTLA4*-modified MSCs group was significantly higher than that in the DBM with control MSCs or DBM alone groups. The trend in β-catenin expression followed that for POSTN (Fig. 4B,C).

### Downregulation of POSTN in immune activation condition *in vitro*

**Figure 5:**
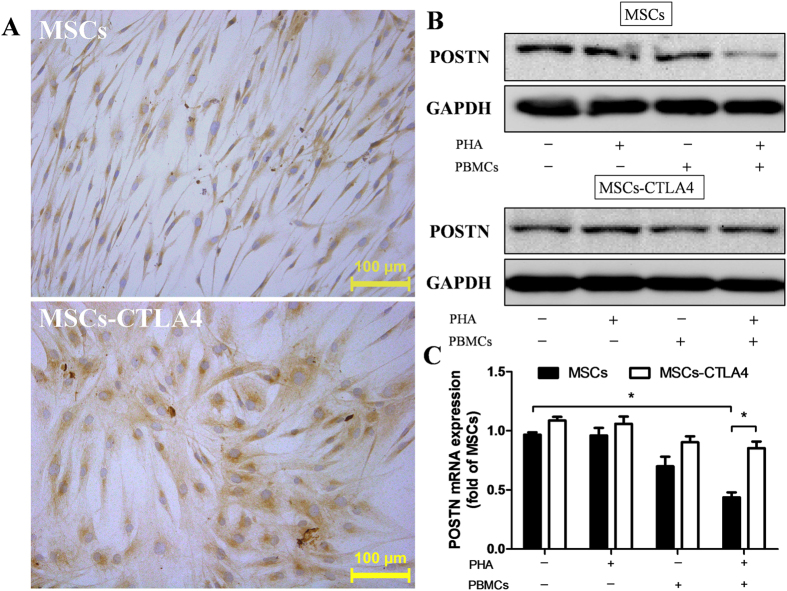
POSTN expression in an immune activation condition *in vitro*. (**A**) The localization of POSTN expression in MSCs and *CTLA4*-modified MSCs was detected by immunohistochemistry. Scale bars: 100 μm. (**B**) MSCs or *CTLA4*-modified MSCs were cultured in the absence (−) or presence (+) of PHA or PBMCs for 3 days as indicated, and POSTN expression was detected by western blotting (20 μg each). (**C**) POSTN mRNA levels were quantified by real-time PCR. n = 3, *P < 0.05.

To simulate the immune rejection culture microenvironment *in vitro*, MSCs or *CTLA4*-modified MSCs were co-cultured with activated peripheral blood mononuclear cells (PBMCs) for 72 h. By immunohistochemistry, POSTN was found mainly located in the cytoplasm of both MSCs and *CTLA4*-modified MSCs (Fig. 5A). Our western blotting and qPCR results showed that POSTN was stably expressed in MSCs and *CTLA4*-modified MSCs treated with PBMCs or phytohemagglutinin (PHA). Under the immune activation condition, POSTN expression in MSCs was down-regulated significantly compared with that in control and *CTLA4*-modified MSCs, and POSTN was still stably expressed in *CTLA4*-modified MSCs (Fig. 5B,C).

### POSTN promoted osteogenic differentiation of MSCs possibly via the Wnt signaling pathway

**Figure 6:**
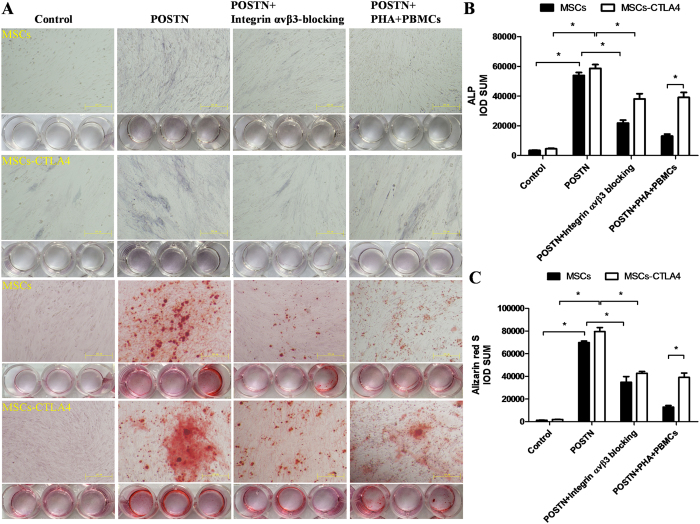
POSTN promoted osteogenic differentiation of CTLA4-modified MSCs through interacting with Wnt signaling pathway. (**A**,**B**) Treatment with soluble POSTN, blockage of integrin αvβ3, and co-cultured with PBMCs and PHA were applied to MSCs and *CTLA4*-modified MSCs as described, and cell lysates (20 μg each) were subjected to western blotting. Untreated MSCs and *CTLA4*-modified MSCs were used as the control. (**C**~**F)** p-LRP 6, GSK-3α/β, β-catenin, and Runx2 expression levels were quantified by ImageJ2x and presented as fold-changes over the control. n = 3, *P < 0.05.

To detect the osteogenesis inductive effect of POSTN in MSCs and *CTLA4*-modified MSCs, the osteogenesis markers such as alkaline phosphatase (ALP) production and calcium nodule formation were detected by Alizarin Red S (ARS) staining (Fig. 6A). The quantitative analysis showed that treatment with POSTN could increase the production of ALP and calcium nodules and blockage of integrin αvβ3 could prevent this increased osteogenic effect in both MSCs and *CTLA4*-modified MSCs. However, under the immune activation condition, the increased osteogenic effect of POSTN was only found in *CTLA4*-modified MSCs and not in MSCs (Fig. 6B,C).

**Figure 7:**
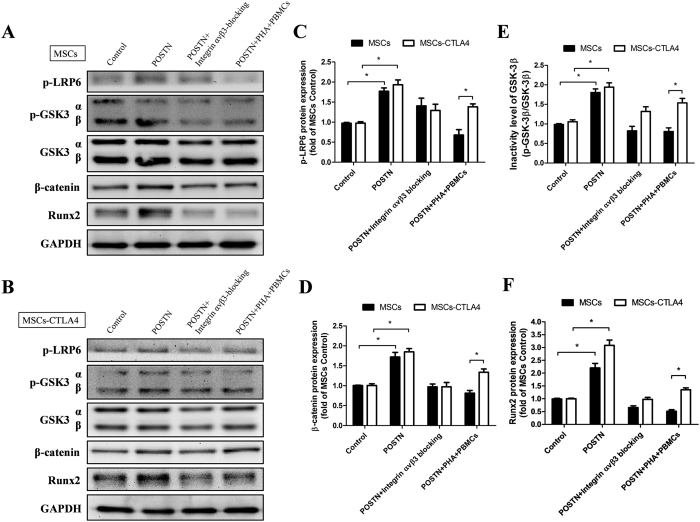
POSTN enhanced the osteogenic differentiation in MSCs and CTLA4-modified MSCs *in vitro* via integrin αvβ3. (**A**) ALP staining was performed after 9 days and Alizarin red S staining was performed after 13 days with treatments as previously described. Untreated MSCs and *CTLA4*-modified MSCs were used as the controls. (**B**,**C**) The IOD of ALP and Alizarin red S was quantified by Image-Pro Plus 6.0. n = 3, *P < 0.05.

To elucidate the mechanism by which POSTN induced osteogenesis, MSCs and *CTLA4*-modified MSCs were treated with soluble POSTN (500 ng/mL, R&D Systems) for 3 days, and the expression of associated proteins was analyzed by western blotting (Fig. 7A,B). Our results showed that after treatment with POSTN, the expression levels of phosphorylated lipoprotein-related protein (p-LRP)-6, active glycogen synthase kinase (GSK)-3β (p-GSK-3β-Ser9/GSK-β), β-catenin, and runt-related transcription factor 2 (Runx2) were significantly increased compared with those in control cells. After blockage of integrin αvβ3, the expression levels of p-LRP-6, inactive GSK-3β, β-catenin, and Runx2 returned to similar those in control cells. Notably, under the immune activation condition, the increased osteogenic effect of POSTN was observed only in *CTLA4*-modified MSCs and not in MSCs (Fig. 7C–F).

## Discussion

The reparative effect of allo-MSC-based bone tissue engineering constructs for large bone defects has been reported, and it is known that the immunosuppressant is necessary for transplantation of allo-MSC-based bone tissue engineering constructs^7^,^8^. Previously, we constructed a bone tissue engineering platform based on MSCs modified to express CTLA4, an important immunosuppressive molecule, and reported the ectopic osteogenic effect of *CTLA4*-modified MSCs within a bone tissue engineering construct^21^,^22^,^23^. In this study, we further explored the *in situ* restoration effect of *CTLA4*-modified MSCs in a rabbit model of a radius defect and discovered that the specific mechanism of *CTLA4*-modified MSC osteogenesis involves interaction between POSTN and the Wnt/β-catenin pathway.

In our previous studies, the ectopic osteogenic effect of *CTLA4*-modified MSCs within a bone tissue engineering platform was demonstrated following subcutaneous implantation or transplantation in femoral muscle bags in rats, but the osteogenesis microenvironment within a bone defect is very different from these environments^21^,^22^,^23^. In this study, we chose the *in situ* critical-sized segmental radius defect rabbit model to investigate the mechanism of the osteogenic effect of *CTLA4*-modified MSCs within a bone tissue engineering platform.

In our study, we found no significant differences between the bone formed after implantation of DBM with unmodified MSCs and implantation of DBM alone. In contrast, the quantity and quality of the regenerated bone tissue after implantation of DBM with *CTLA4*-modified MSCs were significantly higher than that after implantation of DBM with unmodified MSCs or DBM alone. This could be due to the activation of the immune system caused by the allo-MSC implant, which did not favor MSC osteogenesis and instead led to unsatisfactory bone repair effect. These results indicated that without CTLA4, the allo-MSC implant barely improved the bone repair effect of DBM, and expression of CTLA4 can activate an immunosuppressive effect in MSCs to accelerate bone healing significantly.

It has been suggested that activation of the immune system by T cells has a negative impact on the reconstruction of bone within bone defects. Interleukin (IL)-2 produced by activated T cells is a growth factor that mediates the production of interferon (IFN)-γ^24^. Moreover, IFN-γ can not only enhance the immunogenicity of MSCs by elevating the major histocompatibility complex (MHC)-II expression^25^, but also enhance tumor necrosis factor (TNF)-α signaling, resulting in apoptosis of MSCs^26^. As a vital co-inhibitory molecule expressed on activated T cells, CTLA4 can block the B7-CD28 co-stimulatory pathway by competitively inhibiting the CD28 binding with B7 on antigen-presenting cells (APCs) and T-cell activation to induce the immune tolerance^9^. Our previous study demonstrated that CTLA4 down-regulates IL-2 and IFN-γ production under the immune activation condition^10^. Moreover, in this study, we found that POSTN expression was decreased significantly under the immune activation condition, and the presence of CTLA4 maintained POSTN expression in MSCs not only under the immune activation condition *in vitro*, but also in the area of the tissue engineering-based implant *in vivo*. This finding indicated that some factors induced under the immune activation condition can down-regulate POSTN expression, and POSTN may be the key factor for osteogenesis of *CTLA4*-modified MSCs following xenotransplantation in a tissue engineering construct.

With a vital role in cell–ECM interactions, POSTN can bind to integrins αvβ3 and αvβ5 to regulate cell mobility and adhesion^27^. In bone tissue, POSTN is preferentially expressed by periosteal osteoblasts in response to mechanical stimulation or parathyroid hormone (PTH), bone morphogenetic protein (BMP)-2, and transforming growth factor (TGF)-β, and it can regulate the osteogenic differentiation of osteoblasts to enhance bone formation^28^. Our pervious study also confirmed that the activation of erythropoietin-producing hepatocyte receptors (Eph) B4 up-regulates POSTN expression in MSCs and plays a crucial role in regulating bone homeostasis through an unknown mechanism^29^. A recent study reported that POSTN interacts with the Wnt/β-catenin signaling pathway indirectly by inhibiting sclerostin expression in bone^30^. It is known that the canonical Wnt/β-catenin signaling pathway is crucial for bone homeostasis, and the phosphorylation level of β-catenin regulated by GSK-3β is the key junction in the pathway^31^,^32^. β-catenin is known to promote the osteogenic differentiation in MSCs and can be phosphorylated by GSK-3β, which leads to its degradation by proteasomal machinery, and the activity of GSK-3β is negatively correlated with the level of p-GSK-3β-Ser9^33^. Activation of the Wnt/β-catenin signaling pathway occurs via two membrane receptors, including a seven-pass transmembrane receptor Frizzled (FZD) and a single-pass transmembrane co-receptor referred to as LRP-6^34^. Studies have shown that POSTN can decrease the activity level of GSK-3β by increasing the phosphorylation level of LRP-6 to promote cancer cell metastasis^35^,^36^. In this study, we confirmed that the increased phosphorylation of p-LRP-6 and inactivity of GSK-3β (p-GSK-3β-Ser9/GSK-β) caused by POSTN also can be detected in MSCs and *CTLA4*-modified MSCs, as well as the expression and production of β-catenin, Runx2, ALP, and calcium nodules. Our results not only reveal a new mechanism of POSTN activity in osteogenic induction, but also explain why EphB4 was found to interact with the Wnt/β-catenin signaling pathway in our previous study^23^. At present, the exact role of allo-MSCs in this bone tissue engineering strategy remains unclear. Yuan *et al*. reported that the implanted allo-MSCs might not form new bone directly, but may recruit host MSCs to repair the defect through paracrine secretion^37^. Thus, the chemotaxis-related mechanism of POSTN for recruiting host MSCs in the bone tissue engineering strategy employing *CTLA4*-modified MSCs needs to be explored in the future.

In summary, the present study demonstrated that a bone tissue engineering construct containing *CTLA4*-modified MSCs had an excellent reparative effect in large bone defects *in situ* following xenotransplantation, and POSTN expression maintained in the presence of CTLA4 promoted bone repair by up-regulating LRP-6 phosphorylation and activating the Wnt/β-catenin signaling pathway under the immune activation condition. The MSCs used in the present study were pooled from three healthy male donors aged 20–25 years, and our inability to determine the donor-to-donor variability is a limitation of our study. This study not only reveals the osteogenic mechanism of *CTLA4*-modified MSCs within a bone tissue engineering platform but also brings a better understanding of the osteoinductive mechanism of POSTN in maintaining bone homeostasis.

## Methods

### Ethics statement

The study was approved by the Institutional Ethical Committees of Southwest hospital, and informed consent was obtained from donors in accordance with the Declaration of Helsinki. The animal studies were approved by the Animal Ethics Committees of Third Military Medical University. All animals were handled strictly according to the Animal Ethics Procedures and Guidelines of the People’s Republic of China.

### Isolation and culture of human MSCs

MSCs were harvest, isolated, and expanded in culture as previously described^10^. Briefly, approximately 20 ml bone marrow was obtained from three healthy adult donors (males, 20–25 years old) at the Southwest Hospital. The bone marrow samples were diluted in 20 ml phosphate-buffered saline (PBS) and separated in Percoll solution (1.073 g/mL; Pharmacia Corporation) by density gradient centrifugation (900 g for 30 min at 20 °C). The resulting cells were resuspended and cultured at a density of 7.55 × 10^6^ cells per 37.5-cm^2^ flask in human MSC basal medium containing 10% fetal bovine serum (OriCell Human Mesenchymal Stem Cell Growth Medium, Cyagen Biosciences, Guangzhou, China) at 37 °C in 5% CO_2_. The adherent cells were detached using 0.25% trypsin/EDTA (HyClone, Logan, UT, USA) at 90% confluence and replated at a density of 6 × 103/cm^2^. The third passage cells were used for further experiments.

### Infection of human MSCs with adenovirus containing CTLA4-Ig gene

*CTLA4*-modified MSCs were generated as previously described^10^. Briefly, MSCs were infected with recombinant adenoviruses expressing genes encoding CTLA4-Ig with enhanced green fluorescent protein (EGFP) at a titer of 3 × 10^6^ colony forming units (CFUs)/mL. The efficiency of infection was assessed by flow cytometry.

### Establishing the *in vitro* immune activation condition

The immune activation condition was established as previously described^10^. Briefly, PBMCs from the peripheral blood of adult donors were separated by density gradient centrifugation (450 g for 25 min at 20 °C) in Ficoll–Hypaque solution (1.077 g/mL; Sigma-Aldrich) and treated with PHA at 2.5 μg/mL PHA per 1 × 10^6^ PBMCs to establish the immune activation condition *in vitro*. MSCs or *CTLA4*-modified MSCs were added at an MSCs:PBMCs ratio of 1:5 and co-cultured for 72 h.

### Osteogenic differentiation

To induce osteogenic differentiation, the MSC basal medium was replaced with special conditional osteogenic differentiation medium (OriCell MSC Osteogenic Differentiation Medium, Cyagen Biosciences), which was refreshed every 3 days. After 9 days, ALP staining was performed with a FASTBCIP/NBT tablet (Sigma-Aldrich, St. Louis, MO, USA), and after 13 days, calcium nodules were stained by 0.4% ARS staining (Sigma-Aldrich) after 13 days according to the manufacturer’s instructions.

### Real-time polymerase chain reaction (PCR)

Total RNA from cells was extracted using the QIAGEN Rneasy Mini kit (QIAGEN, Hilden, Germany) according to the manufacturer’s instructions and quantified using an ultraviolet spectrophotometer (Beckman Coulter DU-600, Indianapolis, IN, USA). 2 μg cDNA was synthesized using the PrimeScript 1st Strand cDNA Synthesis Kit (TaKaRa, Shiga, Japan), and real-time quantitative PCR was carried out using the 7500qPCR System (Applied Biosystems, Foster City, CA, USA) with SYBR Premix EX Taq II (TaKaRa). Glyceraldehyde-3-phosphate dehydrogenase (GAPDH) was used as a reference gene. The primer sequences were as follows:

POSTN: forward primer, GCCATCACATCGGACATA, and reverse primer, CTCCCATAATAGACTCAGAACA;

GAPDH: forward primer, ACCCATCACCATCTTCCAGGAG, and reverse primer, GAAGGGGCGGAGATGATGAC.

### Western blot analysis

Total protein from cells was prepared in radio immunoprecipitation (RIPA) lysis buffer (KeyGENBioTECH, Beijing, China), and 20 μg protein was separated by 8% sodium dodecyl sulfate (SDS)-polyacrylamide gel electrophoresis (PAGE) and transferred onto polyvinylidene fluoride (PVDF) membranes (Millipore, Billerica, MA, USA). Membranes were blocked in 3% bovine serum albumin (BSA) in Tris-buffered saline (TBS) and incubated overnight at 4 °C with primary antibodies against POSTN (1:1500, Abcam), Runx2, phosphorylated-LRP-6, GSK-3α/β, phosphorylated-GSK-3α/β (Ser21/9), β-catenin (all 1:1000, Cell Signaling Technology, Danvers, MA, USA), and GAPDH (1:12000, Sanjian, Tianjin, China). Membranes were washed with PBST (PBS containing 0.1% Triton X-100) and incubated with horseradish peroxidase (HRP)-conjugated anti-mouse IgG or anti-rabbit IgG (GE Healthcare, Amersham, UK) at a dilution of 1:5000 as a secondary antibody for 1 h at room temperature. The signals were detected using an enhanced chemiluminescence kit (Amersham Biosciences) and ChemiDoc XRS (Bio-Rad). Quantitative analysis was performed using ImageJ2x software (National Institutes of Health, Bethesda, MD, USA).

### Critical-sized radial defect and xenotransplantation of DBM/MSC constructs

The DBM constructs were fabricated as previously described^23^. Eighteen New Zealand male rabbits were randomly divided into three groups implanted with: (1) DBM only (n = 6); (2) DBM with unmodified MSCs (n = 6) and (3) DBM with *CTLA4*-modified MSCs (n = 6). All rabbits were anesthetized by intravenous injection of 3% pentobarbital sodium (30 mg/kg), and a unilateral segment of the periosteum and radius with a critical-sized length of 2.5 cm was excised using a circular saw. Then, the defects were filled with DBM, DBM with MSCs, or DBM with *CTLA4*-modified MSCs as described above, and the incision was sutured.

### X-ray and micro-CT examination

Plain X-ray images of forearm radius defects were taken at 4 and 8 weeks after implantation. All the radius and ulnas were harvested of 8 weeks post-implantation for micro-CT scanning (Quantum FX micro-CT Imaging System, PerkinElmer, MA, USA) with the following parameters: X-ray voltage, 90 kV; 160 μA; 20-mm pixel size. Three-dimensional images were reconstructed in the region of interest (ROI) that was set as a cylinder at the center of each radius defect. The morphometric indices including bone mineral density (mg/cc), bone volume (mm^3^), and bone volume fraction (bone volume/tissue volume ratio, BV/TV) were assessed.

### Histological evaluation

At 2 months post-implantation, radii with treated defects (n = 6/group) were fixed in 4% paraformaldehyde for 10 days and decalcified in 10% ethylenediaminetetraacetic acid (EDTA) for 30 days. Then the samples were embedded in paraffin and sectioned at 5 mm. H&E and Masson’s Trichrome staining were performed to assay the tissue morphology.

### Immunohistochemistry

Immunohistochemistry was performed using SABC IHC kits (Zhongshan Corporation) with primary antibodies to POSTN and β-catenin (1:500, Santa Cruz Biotechnology, Santa Cruz, CA, USA) according to the manufacturer’s instructions, and nuclei were counterstained with hematoxylin. Images were captured using a Leica Microsystems microscope (DFC300 FX, Heerbrugg, Switzerland). The brief procedure for integrated optical density (IOD) analysis was performed as follows: a ROI for a positively stained area was analyzed, and the average signaling intensity was quantified by Image-Pro Plus 6.0.

### Statistical analysis

Data are presented as mean ± standard error of the mean (SEM). Statistical analysis was performed with two-way analysis of variance (ANOVA) using the GraphPad Prism 5.0 statistical software package (GraphPad, La Jolla, CA, USA). P < 0.05 was considered statistically significant.

## Additional Information

**How to cite this article**: Zhang, F. *et al*. Periostin Upregulates Wnt/β-Catenin Signaling to Promote the Osteogenesis of *CTLA4*-Modified Human Bone Marrow-Mesenchymal Stem Cells. *Sci. Rep.* **7**, 41634; doi: 10.1038/srep41634 (2017).

**Publisher's note:** Springer Nature remains neutral with regard to jurisdictional claims in published maps and institutional affiliations.
