# Author Correction: Periostin Upregulates Wnt/β-Catenin Signaling to Promote the Osteogenesis of *CTLA4*-Modified Human Bone Marrow-Mesenchymal Stem Cells

**DOI:** 10.1038/s41598-021-89264-7

**Published:** 2021-04-29

**Authors:** Fei Zhang, Keyu Luo, Zhigang Rong, Zhengdong Wang, Fei Luo, Zehua Zhang, Dong Sun, Shiwu Dong, Jianzhong Xu, Fei Dai

**Affiliations:** 1 Department of Orthopaedics, National & Regional United Engineering Laboratory, Southwest Hospital, Third Military Medical University, Chongqing, China; 2 Department of Biomedical Materials Science, School of Biomedical Engineering, Third Military Medical University, Chongqing, China

Correction to: *Scientific Reports* 10.1038/srep41634, published online 27 January 2017

This Article contains an error in Figure 4 where the immunohistochemistry photo of POSTN for DBM group was incorrect. The correct Figure 4 appears below as Figure 1.

**Figure 1:**
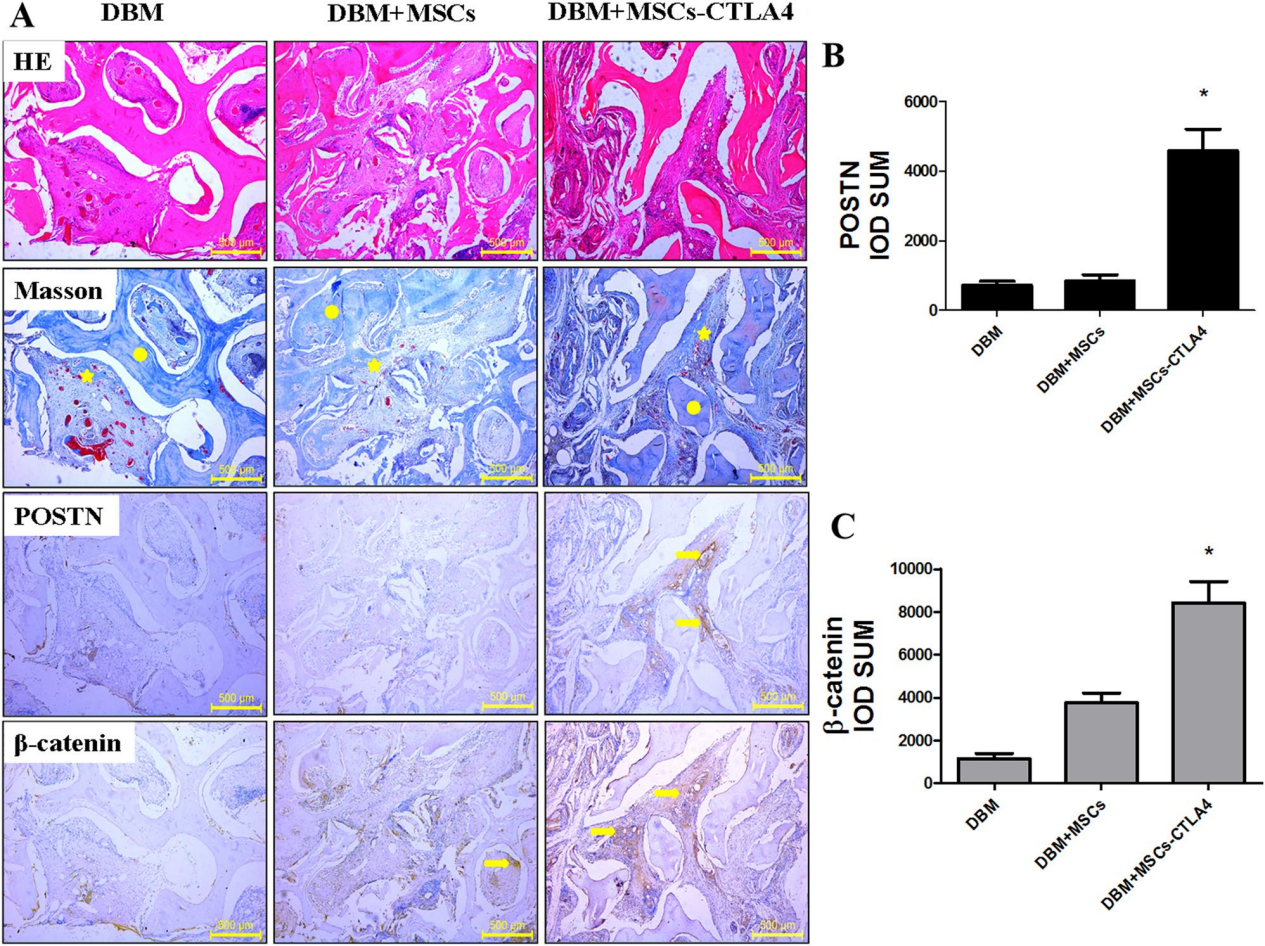
(**A**) Histological analysis by H&E and Masson’s Trichrome staining (circle: DBM; pentagram: new bone formation area) of newly formed bone, and the expressions of POSTN and β-catenin detected by immunohistochemistry at 2 months post implantation (arrow: positive expression). Scale bars: 500 μm. (**B**,**C**) The IOD of POSTN and β-catenin was quantified by Image-Pro Plus 6.0. n = 3, *P < 0.05.

